# Resolving missing protein problems using functional class scoring

**DOI:** 10.1038/s41598-022-15314-3

**Published:** 2022-07-05

**Authors:** Bertrand Jern Han Wong, Weijia Kong, Limsoon Wong, Wilson Wen Bin Goh

**Affiliations:** 1 School of Biological Sciences, Nanyang Technological University, 60 Nanyang Drive, Singapore, 637551 Singapore; 2 School of Computing, National University of Singapore, Singapore, Singapore; 3 Lee Kong Chian School of Medicine, Nanyang Technological University, 60 Nanyang Drive, Singapore, 636921 Singapore; 4 Center for Biomedical Informatics, Nanyang Technological University, Singapore, Singapore

**Keywords:** Network topology, Proteome informatics, Statistical methods

## Abstract

Despite technological advances in proteomics, incomplete coverage and inconsistency issues persist, resulting in “data holes”. These data holes cause the missing protein problem (MPP), where relevant proteins are persistently unobserved, or sporadically observed across samples, hindering biomarker discovery and proper functional characterization. Network-based approaches can provide powerful solutions for resolving these issues. Functional Class Scoring (FCS) is one such method that uses protein complex information to recover missing proteins with weak support. However, FCS has not been evaluated on more recent proteomic technologies with higher coverage, and there is no clear way to evaluate its performance. To address these issues, we devised a more rigorous evaluation schema based on cross-verification between technical replicates and evaluated its performance on data acquired under recent Data-Independent Acquisition (DIA) technologies (viz. SWATH). Although cross-replicate examination reveals some inconsistencies amongst same-class samples, tissue-differentiating signal is nonetheless strongly conserved, confirming that FCS selects for biologically meaningful networks. We also report that predicted missing proteins are statistically significant based on FCS *p* values. Despite limited cross-replicate verification rates, the predicted missing proteins as a whole have higher peptide support than non-predicted proteins. FCS also predicts missing proteins that are often lost due to weak specific peptide support.

## Introduction

Proteomic approaches are a powerful means of interrogating the phenotype of complex biological samples. Recent advances in hardware have made proteomics increasingly useful for clinical investigation. These include more efficient protein extraction procedures (e.g. PCT^1^), brute-force spectra-capture methods (e.g. Sequential Window Acquisition of all THeoretical Spectra; SWATH^2^), and improved multiplexing kits^3^. Although proteomics generally falls behind genomics on throughput and adoption, developments in proteomic technologies are of critical importance to biological and clinical/translational research; assaying protein identities/quantities, and their associated post-translational modifications, paints an immediate picture of the underlying functional landscape—a crucial limitation that genomics technologies cannot readily address.

Despite recent technological improvements, current proteomics still suffers from incomplete proteome coverage issues (not all proteins in an organism are observed in a single screen), and more critically, inconsistency issues (different screens on the same samples generate different protein sets)^4^. These issues give rise to problems during functional analysis, impeding efforts towards understanding the functional phenotype, unravelling mechanisms, or identifying reproducible biomarkers.

Network-based approaches provide an interesting avenue towards resolving these challenges, by representing proteomic samples in terms of their functional modules and pathways, perhaps overcoming extensively heterogenous proteome coverage and limited technical reproducibility. Previous efforts have shown that network-based analyses permit a deeper understanding of biological mechanisms^5^ and facilitate reproducible comparative analyses^6–8^. Several network-based analyses methods exist; among these, Functional Class Scoring (FCS) is a notable example that was previously shown to outperform other network methods^9,10^ (see Supplementary Discussion for a more detailed overview of network-centric approaches).

To further demonstrate the strength and relevance of network-based methods for proteome profiling and functional analysis, we provide an update on FCS benchmarking and its performance on proteomic data generated with more recent technologies such as Data Independent Acquisition (DIA)/SWATH. Additionally, we develop a more rigorous method of evaluating validity and benchmarking FCS-based missing protein prediction, utilizing cross-verification of technical replicates; this study places particular focus on the challenging tasks of recovering low-support or low-abundance proteins (e.g., proteins not meeting the two-peptide rule, or supported by peptides with low intensities).

## Materials and methods

All methods were performed in accordance with the relevant guidelines and regulations.

### Renal cancer control (RCC)

The renal cancer control dataset (RCC), from the study of Guo et al.^1^, comprises 12 SWATH runs originating from a normal human kidney test tissue digested in quadruplicates (× 4) and each digest analyzed in triplicates (× 3) using a tripleTOF 5600 mass spectrometer (AB Sciex).

**Figure 1:**
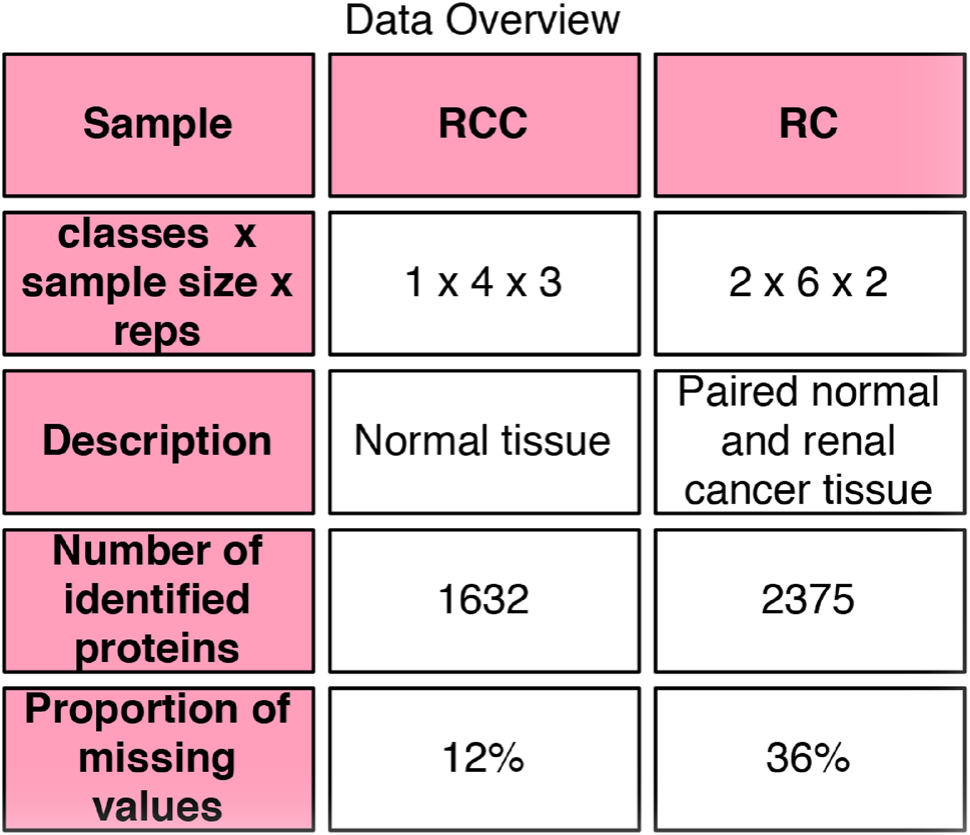
Broad overview of the dataset (Renal Cancer, RC; Renal Cancer Control, RCC). It is noteworthy that there are many more data holes, and therefore missing proteins, in RC than in RCC.

1,632 proteins are quantified across the 12 SWATH maps with a peptide and protein false-discovery rate (FDR) of 1%. General details of RCC are shown in Fig. 1. The RCC dataset is less complex (one phenotype class, and no inter-individual variability), but also less data holes (12% missing values), suggesting less inconsistency between samples.

### Renal cancer (RC)

The renal cancer (RC) dataset from Guo et al*.*^1^ comprises 24 SWATH runs originating from six pairs of non-tumorous and tumorous clear-cell renal carcinoma tissues, with two technical replicates (duplicates) each (Fig. 1).

All SWATH maps are analyzed using OpenSWATH^11^ against a spectral library containing 49,959 reference spectra for 41,542 proteotypic peptides from 4624 reviewed SwissProt proteins. The library is compiled via library search of spectra captured in DDA mode (linking spectra m/z and rt coordinates to a library peptide). Protein isoforms and protein groups are excluded from this analysis. Proteins are quantified based on the intensities of the top two most abundant peptides.

Further details on the RC and RCC datasets are described in the NetProt package^12^.

### Colorectal cancer (CR)

To demonstrate that the significant complexes (e.g. via FCS), and by implication, their associated proteins, present in a sample tend to be more similar if they come from the same tissue (in spite of different proteomics screen), we include a non-kidney dataset (based on 30 normal liver samples from the colorectal dataset of Zhang et al*.*^13,14^) to be compared against RC and RCC.

### Network feature vector based on real biological complexes

Biological complexes can recover missing proteins with unmatched sensitivity, and are more effective and practical for analytical use than inferred clusters from protein interaction networks^7–9^ : Firstly, information on biological complexes tends to be highly centralized and easily accessible, e.g. the CORUM database^15^ for human complexes and MIPS^16^ for yeast. Secondly, biological complexes exhibit high signal enrichment^17^, over many other sources of data, including expressional correlation and predicted subnetworks. Third, a set of complexes can be a standardizable reference, facilitating cross-comparability between different studies. Finally, standardizing complex representation is easy: A complex is simply a list of its constituent proteins (where stable identifiers for proteins, e.g., UniProtKB accessions, already exist); and information regarding the exact topological configuration amongst constituent proteins in a complex is not required (except when we want to distinguish between core/peripheral proteins, or classify complexes into families). For our network feature vector, we use curated protein complexes from the CORUM database (release 2018)^15^. As small complexes can cause high fluctuation in test statistics^18,19^, only protein complexes of at least size 5 are used in analysis (600 out of 1,323).

### Functional Class Scoring (FCS)

In FCS^9^, given a set of observed proteins in a proteomics screen S, and a list of component proteins *M*_*i*_ from a real protein complex *C*_*i*_ (where *C*_1_ … *C*_*n*_ constitute the list of protein complexes in the complex vector), an observed overlap, *O*_*i*_ is expressed as:

$${O}_{i}=\frac{|S\cap {M}_{i}|}{|{M}_{i}|}$$

To determine if this overlap *O*_*i*_ is significant, 1,000 randomized complexes of size |*M*_*i*_| (i.e., the size of *C*_*i*_) are generated using a reference pool of unique proteins drawn from the complexes *C*_*i*_ … *C*_*n*_. From the 1,000 randomized complexes, a vector of null overlaps, *N*_1_ … *N*_1000_ is generated. E.g., for the *j*-th randomized complex, which comprises the set of proteins *K*_*j*_, we may calculate a null overlap *N*_*j*_, by comparing *K*_*j*_ against *S*.

$${N}_{j}=\frac{|S\cap {K}_{j}|}{|{K}_{j}|}$$

**Figure 2:**
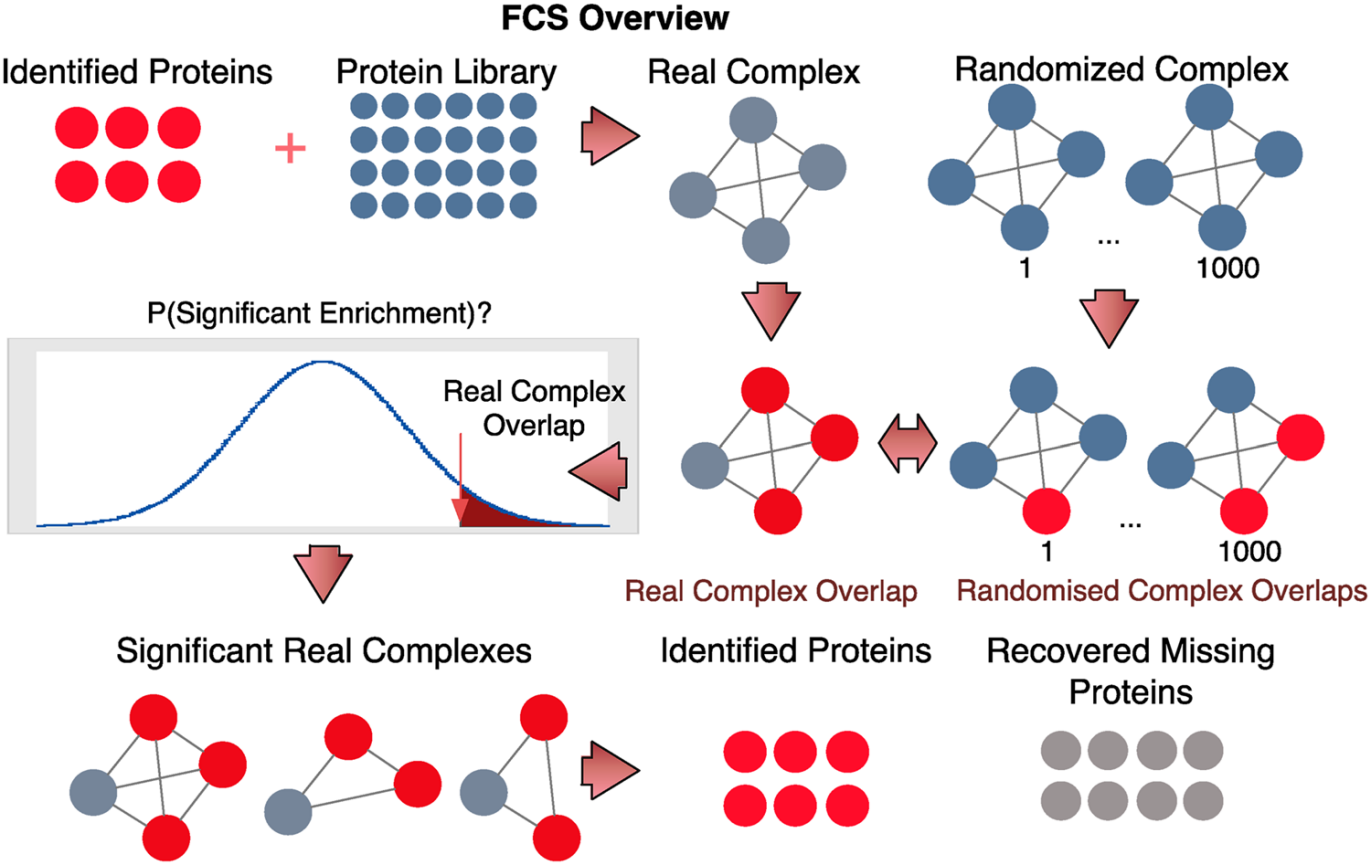
How Functional Class Scoring (FCS) works. In FCS, given a set of observed proteins and a real protein complex, an observed overlap is defined. To determine if this observed overlap is significant, randomized complexes equal to the size of the original complex are generated 1,000 times (using a pool of proteins found in complexes), and a null overlap calculated each time. The null overlaps form a null distribution allowing an empirical p-value to be defined as the proportion of null overlaps greater than or equal to the observed overlap.

The empirical p-value is the proportion of null overlaps in *N*_1_ … *N*_1000_ greater than or equal to the observed overlap *O*_*i*_ (Fig. 2). For the *i*-th complex *C*_*i*_ in the complex vector, its p-value, *pval*_*i*_ is:

$${pval}_{i}=\frac{\sum_{j=1}^{1000}\,if \,{N}_{j}\ge {O}_{i}then \,1 \,else \,0}{1000}$$

If *pval*_*i*_ falls below 0.05, the complex *C*_*i*_ is statistically significant. Given the set *M*_*i*_ of proteins in *C*_*i*_, and the set *S* of observed proteins, the set of missing proteins that are predicted to be present is defined as *M*_*i*_ \*S*.

### Evaluation of missing protein recovery

Predicted missing proteins are verified based on several scenarios: (i) proteins corresponding to the peptide list consisting of all significant peptide-spectra matches (PSMs) from the sample itself, (ii) proteins corresponding to the peptide list consisting of all significant PSMs from the cross-batch replicate, and (iii) proteins corresponding to the union of the PSMs from self and cross-batch replicate. Additionally, we also consider the following naïve scenarios: (iv) Observed proteins in the cross-batch replicate, and (v) proteins corresponding to FCS-significant complexes in the cross-batch replicate.

To determine whether the total set of recovered proteins is significant, we assume that cross-batch replicates should report the same proteins (In practice they do not, thus leading to the MPP). We run FCS on one replicate, and test whether the missing proteins that are predicted to be present show up in other replicates. Let *R* be the set of missing proteins predicted to be present, and r be the members of *R* that show up in other replicates. We generate a random set R’ of the same size as *R* and let *r’* be the members of *R’* that show up in other replicates. This randomization is repeated many times, and we determine whether |r|/|R| lie at the extreme right end of the |*r’*|/|*R’*| null distribution. If so, we say that this set of recovered proteins is significant and relevant towards the samples being studied.

When comparing for overlaps, e.g., to evaluate whether similar missing proteins are predicted across different samples, we use the Jaccard index. Given two sets *X* and *Y*, we may define the Jaccard Index *J*(*X*,*Y*) as:

$$J\left(X,Y\right)=\frac{X\cap Y}{X\cup Y}$$

## Results

### Missing values are widespread amongst analyzed samples

**Figure 3:**
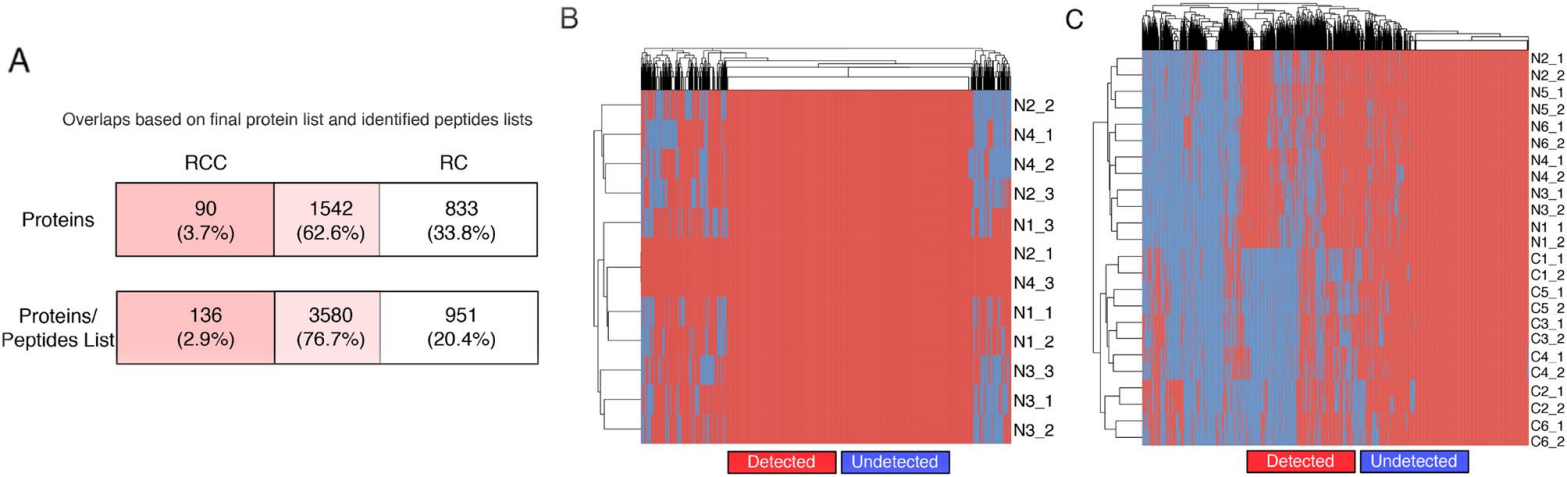
Characteristics of analyzed datasets. (**A**) Protein and peptide agreements between RC and RCC. Each row shows proteins unique to RCC (dark pink), shared between RCC and RC (light pink) and unique to RC (white). The top row shows only proteins in the finalized observed protein list while the bottom includes all proteins with at least one unique peptide. (**B**)**:** Distribution of detected and missing proteins in RCC. Hierarchical clustering suggests that the distribution of data holes across samples is not batch related (samples are named by Class/Biological Replicate/Technical Replicate, e.g., N2_2 means "Normal” class sample 2, batch 2). (**C**): Distribution of detected and missing proteins in RC. Hierarchical clustering suggests that the distribution of data holes across samples is not batch related (Class/Biological Replicate/Technical Replicate). While approximately 20% proteins are consistently observable across samples, most proteins have missing values meaning they are not observed in a subset of samples. Proteins more prone to exhibit missing behavior cannot be solely explained by low abundance (cf. Supplementary Fig. 1).

The renal cancer dataset RC is more complex than the single-tissue benchmark dataset RCC. This is because RC comprises two phenotype classes, has higher individual variability (due to more patients), and ~ 3 × more data holes (36%). Although many proteins observed in RC and RCC are shared (Fig. 3A), a quick check on the dispersal of missing proteins across samples in RC also indicates that the missing proteins are dispersed across a wider range of proteins (Fig. 3B and C).

As we are interested in assessing missing protein recovery across technical replicates, it is important that batch effects do not dominate outcome^20^. Figure 3B/C show the relationships between samples annotated by class and batch (The naming nomenclature is class_sample number_batch; e.g. N2_2 means “Normal” sample 2, batch 2) where we ascertained no obvious batch effects, i.e., the sample do not group broadly by the batch labels .

### FCS-predicted complexes are tissue specific and biologically relevant

While FCS allows us to represent each sample in terms of its statistically significant networks or protein complexes, we next check if these representations are biologically meaningful.

**Figure 4:**
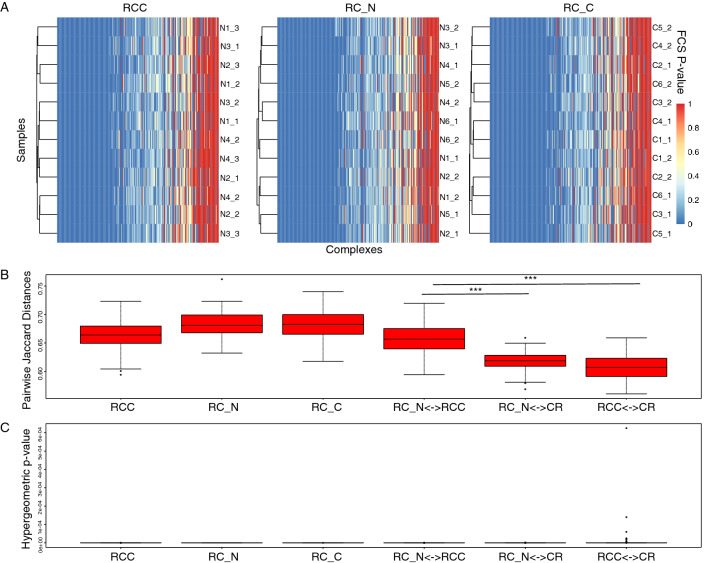
FCS-significant complexes are tissue-specific. (**A**): Distribution of FCS p-values across samples for renal cancer control (RCC) and renal cancer normal (RC_N) and cancer (RC_C) tissues. Even in the same tissue class, individual samples report variable complexes as significant. (**B**) Pairwise tissue similarity based on overlaps of significant complexes. Samples from same tissues class tend to exhibit higher levels of shared complexes (Pairwise Jaccard Index) even when the observed proteins are different. Cross-comparisons (as indicated by a bi-directional arrow, <—>) with a second tissue type (Colorectal; CR tissue) shows significantly lower Jaccard indices (*t*-test; *p* << 0.01 ***). (**C**): Many complexes are shared between different tissues. Despite the significantly lowered Jaccard indices indicating lower significant complex agreement, a large proportion of complexes are still shared amongst different tissues resulting in generally low hypergeometric p-values. However, unshared complexes and their constituent proteins appear to exhibit high tissue-specificity (cf. Supplementary Fig. 2).

We first considered the distribution of FCS p-values for each protein complex across samples in RCC, and the two sample classes of RC (RC_N and RC_C, where N and C refers to normal and cancer classes respectively); cf. Figure 4A. Although many significant complexes are shared amongst samples (blue zones), there is a high degree of heterogeneity, suggesting that FCS across the different samples predicts a notable proportion of different complexes even though they belong to the same class.

Despite this apparent heterogeneity amongst same-class samples, we are curious whether there is conserved signal amongst significant complexes (FCS p-value below 0.05) reported in the same tissue-type, despite different proteomics screens. Based on the inter-sample agreement for RCC, RC_N and RC_C, we find that the Jaccard indices are relatively high (~ 0.65 to 0.70), compared to overlaps against significant complexes derived from another tissue (colorectal in this case); cf. Fig. 4B. Although overlaps fall when we consider similarity of significant complexes between RCC and RC_N (RC_N <-> RCC), the Jaccard indices are still appreciably higher (*p* << 0.01; ***) than when we compare RC_N to CR (RC_N <-> CR) and RCC to CR (RCC <-> CR) (Fig. 4B), suggesting that despite the apparent heterogeneity (in terms of significant complex agreements) amongst same-class samples, there is conserved signal amongst samples derived from the same tissue type, even across different proteomics screens (as with RCC and RC_N).

For each complex overlap between sample pairs, we may also determine a significance measure based on the hypergeometric *p* value (Fig. 4C). Here, regardless of same tissue on same proteomics screen, same tissue on different proteomics screen, or cross-tissue on different proteomics screen, the hypergeometric p-values are all generally low (*p* << 0.01). We speculate this is due to high numbers of shared complexes (e.g., housekeepers—transcriptional, translational and protein degradation machinery, etc.) common to many different tissue types anyway (Supplementary Fig. 2). However, it is noteworthy that the p-values for cross-tissue comparisons appear somewhat less significant, possibly due to lower inter-tissue overlaps (Fig. 4C).

The proteins corresponding to significant complexes unique to liver and kidney may be tissue discriminatory: Based on the Fragments Per Kilobase Million(FPKM) normalized transcriptome profile across 14 different tissues (from the Human BodyMap 2.0; http://www.ebi.ac.uk/arrayexpress/experiments/E-MTAB-513/)^21–23^, we examined gene expressions corresponding to proteins from significant complexes common to RC_N and CR, and proteins from significant complexes unique to RC_N, and proteins from significant complexes unique to CR. The genes are clustered based on hierarchical clustering (Euclidean distance; average linkage). It appears that when examining shared genes encoding proteins belonging to common complexes, kidney and liver are closely spaced amongst the various tissue types but when considering unique genes encoding for proteins belonging to tissue-specific complexes, the liver and kidney tissues are more widely spaced apart (Supplementary Fig. 2). This observation, together with the earlier observation that significant complexes are conserved with respect to tissue type, suggest that predictions made by FCS are biologically relevant, in line with the biological characteristics of the underlying tissue class.

### FCS-based cross-examination of technical replicates yields modest recovery of missing proteins

**Figure 5:**
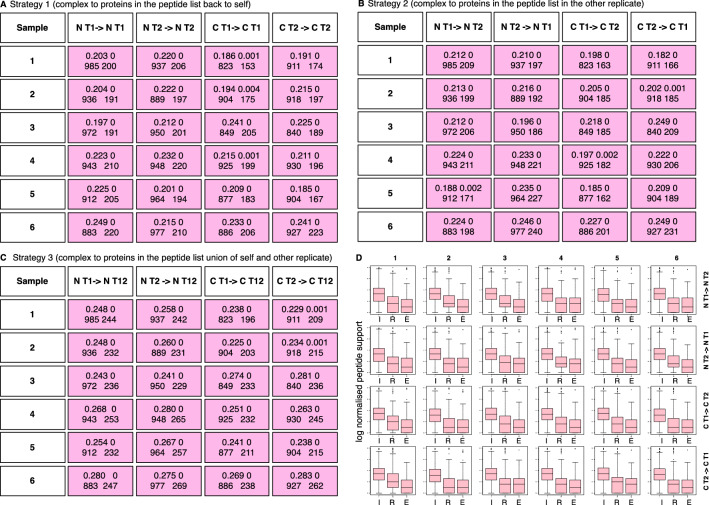
(**A**)**:** Missing-protein verification for RC based on various strategies. A: Missing protein verification based on full peptide list of self. The predicted missing proteins are verified on a peptide list originating from the sample itself (The notation N T1—> N T1 refers to a normal class sample of technical replicate 1 on the left side. The arrow sign is the direction of verification and the N T1 on the right side refers to the peptide list from N1 T1 itself). The four elements in each box are the overlaps and its corresponding *p* value on the top left and right. The bottom left and right are the total number of predicted missing proteins and the number of verified missing proteins. Significant verifications (*p* value ≤ 0.05) are shaded in pink. (**B**)**:** Missing-protein verification based on full peptide list of second technical replicate. Like **A**, but the missing protein verification is performed on the second technical replicate instead. Verification rates are slightly higher but as with (**A**), all verification rates are significant. (**C**): Missing-protein verification based on union of full peptide list from first and second technical replicates. Taking the union of the peptide lists increases verification rates from ~ 20% to ~ 25% but this is still relatively low. Most missing proteins cannot be verified in this direct manner. (**D**): Peptide support across various protein categories (I; Identified refers to observed proteins in the proteomics screen, R; Recovered refers to predicted missing proteins, E; External refers to proteins that are neither observed nor predicted as present in the cross-batch replicate). Identified (Observed) proteins tend to have higher peptide support across the board compared to predicted missing proteins. Unreported proteins that are not predicted as missing and are not observed in the cross-batch replicate tend to have lowest peptide support generally.

Following FCS, we determined the extent and significance of recovery based on verification on three strategies: Based on the set of proteins corresponding to all significant PSMs in the same sample (Fig. 5A), on the set of proteins corresponding to all significant PSMs in the cross-batch replicate (Fig. 5B), and on the union of the set of proteins corresponding to all significant PSMs in the same sample and cross-batch replicate (Fig. 5C). The notation in Fig. 5, e.g., N T1 -> N T2, means N for normal, T is for technical replicate, the direction of the arrow means we are comparing the proteins recovered based on the significant complexes from sample N T1, and checking them against the proteins identified in N T2. We consider each sample from patient samples 1 to 6 separately. The results in each cell of Fig. 5 are shown as two rows: the top row shows the overlap |*r*|/|*R*| and its associated p-value on the left and right respectively (see “Materials and Methods”). The bottom row shows the total number of predicted missing proteins and the number of verified missing proteins on the left and right respectively.

As additional comparisons, we also verify based on observed proteins (i.e., the finalized set of proteins reported in the proteomics screen for a given sample) in the cross-batch replicate (Supplementary Fig. 3A) and verification based on the proteins from significant complexes in the cross-batch replicate (Supplementary Fig. 3B). It is useful to discuss these two naïve scenarios first: In the former, recovery is extremely low. Not all recoveries are statistically significant, and verification rate is around 2 to 5% (Supplementary Fig. 3A). On the other hand, in the latter where we compare missing proteins predicted to be present in one replicate against the FCS-significant complexes in the corresponding cross-batch replicate, the overlap shoots up dramatically to ~ 90% (Supplementary Fig. 3B). Although cross-batch replicates do not report the same protein sets, these proteins nonetheless map back generally to the same protein complexes in the same sample. However, both of these recovery verification methods are not robust: In the former, the verification rate is too low to be useful. This is not surprising; otherwise, taking multiple technical replicates would have easily resolved the MPP. Unfortunately, this suggests that missing proteins tend to be harder to observe/recover generally (see next section). In the latter scenario, we focused on direct verification of significant protein complexes between cross batches, and not on mutually supportive predictions of proteins that are undetected but present in the sample. While naïve, this approach tells us that, despite the different reported proteins between technical replicates of the same samples, we still predict similar complexes. Nevertheless, while some biological signal is evidently conserved, replicates from different still report quite a lot of different significant complexes which may not be meaningful (see again heterogeneity in Fig. 4A).

For verification of predictions of undetected-but-present proteins, the PSM list (where proteins with at least one representative peptide are listed) is used for determining whether there is evidence that a predicted missing protein is indeed present. Interestingly, despite the differences in observed proteins, self-recovery and cross-batch replicate recovery have similar results of ~ 20% recovery rate (Figs. 5A and B), although cross-batch replicate recovery rates are slightly higher.

Taking the union of the PSM lists from self and cross-batch replicate increases verification rates modestly from ~ 20% to ~ 25%; cf. Figure 5C. Although this gives rise to an appreciable improvement of 25% (i.e., 25–20 over 20), verification rates are still low. Apparently, where RC is concerned, most predicted missing proteins (~ 75%) cannot be verified in this manner due to the lack of supporting PSMs. However, given more technical replicates and more support, it is likely possible to improve recovery beyond 25% (as less detectable peptides become observable with increased depth), although we are uncertain if recovery rates are robustly predicted as a function of replicate size, or whether recovery proportion predicted on one dataset is generalizable to other tissues/datasets.

### Peptide support is a stronger contributing component towards missing proteins than low abundance

Low abundance is frequently cited as a cause for missing proteins^24^. A reason for this may be the semi-stochastic loss of proteins in Data-Dependent Acquisition (DDA) paradigm proteomics screens where smaller signals corresponding to low abundance are more likely overlooked. However, low abundance cannot be attributed as a strong or sole contributing factor for the missing proteins observed in the RC dataset, where even at higher abundance levels, missing proteins exist nonetheless (Supplementary Fig. 1A). Moreover, for relatively high-abundance proteins (greater than the median expression level), there does not appear to be any difference for missing values below or above the median missing-value level (Supplementary Fig. 1A). Hence, an alternative explanation is needed to better understand why missing proteins occur.

Under the Data Independent Acquisition (DIA)-derived paradigm, there is no semi-stochastic preselection of precursor peptides based on signal intensity, all spectra are captured if it falls within detection limit. The lack of association between low-abundance proteins and increased missing values (see again Supplementary Fig. 1A) is consistent with the nature of DIA, and perhaps is an artifact associated with the older DDA paradigm (higher-intensity precursor spectra tends to be selected for identification, creating the correlation between low abundance and non-detection). Instead, in DIA, we find that low-confidence PSMs and low peptide support for proteins are generally stronger contributing factors towards MPP. Figure 5D shows the distributions of peptide support for Internal (Observed proteins), Recovered (Verified proteins) and External (Proteins that were neither observed nor predicted to be missing in the cross-batch replicate). Observed proteins tend to have the highest peptide support while predicted missing proteins (expected to be present), has relatively lower peptide support. However, unpredicted proteins not observed in the cross-batch replicate have the least peptide support. It is plausible that proteins with lower peptide support may not consistently meet the statistical threshold required when converting PSMs (based on peptides) to the finalized observed protein list, and this leads towards MPP (and data holes in the observed protein expression matrix).

This is encouraging for the use of complex-based missing protein prediction, since the recovered proteins based on significant complexes are more enriched for higher peptide support than those not predicted to be recoverable at all.

### Unverified predicted missing proteins may not exist in tissue in first place

It is possible that complexes that share many constituent proteins contribute to the high number of non-verifiable proteins reported by FCS. As CORUM is a manually curated database concerned with the annotation of biologically relevant complexes, there is some level of degeneracy associated with complexes of high overlap, which exist in distinct biological niches. For example, the chromatin remodelling complexes nBAF and npBAF have many similar components but are found in different tissues^25^. Consequently, condensing complexes into “super-complexes” based on the superset of their shared components does not produce biologically meaningful entities^26^.

Without tissue-specific information allowing us to only consider kidney tissue-specific complexes, we proposed a simple heuristic: as we believe that since most proteins at the smallest FCS *p* values are considered important, it is possible that tissue specificity (of complexes) may contribute towards some degree of non-verification (i.e., we are considering irrelevant complexes that are significant because of deep sharing of core proteins with a tissue-specific relevant complex). If true, then it is likely that the recovery rates above are severely underestimated due to tissue-specificity issues.

Using sample N1 and its peptide list derived from both its technical replicates as an example, we found a total of 62 unverified proteins, and 557 (observed + verified) proteins. We mapped each of the 62 unverified proteins to the largest complex it is a component of and generated an observed overlap with median of 0.32. Given 1,000 randomized median overlaps, only 7 times were the randomized medians greater, yielding an empirical *p-*value of 0.007, indicating strong support for enrichment of observed + verified proteins in the complexes where the unverified proteins are found. Running the same test on sample N2 also reveals similar results with a *p-*value of 0.008. Hence, it remains possible that these unverified proteins belong to some tissue-specific complex variant absent in the tissue sample. Consequently, incorporating all complexes simultaneously without regard for their tissue specificity or the presence of other same complex family members likely gives rise to a large proportion of unverified proteins. Furthermore, we may be underestimating the verification rates of our predicted missing proteins, due to spurious prediction of proteins that are not expected to be in the tissue altogether. This may necessitate further work towards defining tissue-specific complexomes for more powerful network analytics.

## Discussion

### FCS-significant complexes are biologically meaningful

Although we reported previously that FCS predicted MPs have the highest recovery rates against other network methods^9^, we have not evaluated its use as a single sample profiling method, including whether reported complexes are tissue-specific or idiosyncratic. Here, we demonstrated that samples from the same tissue class exhibit higher levels of shared complexes. Impressively, tissue similarity is strongly conserved despite extensive heterogeneity in the underlying proteomic screen, highlighting the power of network-based methods of protein recovery. Correlations within same-class samples are similarly high. Despite the significantly lower Jaccard indices indicating lower significant complex agreement, a large proportion of complexes are still shared amongst different tissues resulting in generally low hypergeometric p-values. However, unshared complexes and their constituent proteins appear to exhibit high tissue-specificity.

### Tissue-specificity amongst complexes necessitates the development of complex families as a refinement for FCS

We further highlighted the limit to the proportion of verifiable missing proteins. Aside from the lack of any PSMs for unverified proteins, another contributing reason may be biological: Some complexes share common components with one another (the core proteins) and differ by a few peripheral proteins. The peripheral proteins modulate the function of the complex, and in some cases, lead to tissue differentiation. Thus, certain complex forms are found only in certain tissues or cell types ^22^. In other words, there remains a key question of capturing protein complex modularity that the base complexome is unable to address, which would be interesting to examine further at depth.

An example of such tissue-specific modular complexes is the BAF (BRG1-associated factors) family of complexes, which acts as a switch in neuronal differentiation, exists in two forms; neural progenitor (npBAF) and matured (nBAF)^27^. As neural progenitor cells exit mitosis and differentiate into neurons, npBAF complexes, which contain ACTL6a and PHF10, are exchanged for ACTL6b and DPF1/DPF3 subunits in nBAF^27,28^. While the core remains constant, the exchange of peripheral proteins is involved in differentiation, and so, cannot be present together in the same tissue. In FCS, there is no distinction between core and peripheral proteins; supposing there is a family of complexes with a ‘common core’ of proteins, all complexes with sufficient baseline support will be reported as significant, which leads to prediction of peripheral proteins across this family. However, as not all the complexes in the family can form in the same tissue, we should expect that some of the predicted missing proteins to be non-verifiable (the predicted complex does not form in this tissue; and so, its peripheral proteins do not exist in this tissue as well). Consequently, many unverifiable protein predictions may arise from tissue-specificity issues, where the entire complex being considered does not exist in that tissue. It would therefore be of great utility to classify complexes into tissue-specific families, so that we can avoid considering redundant complexes in a tissue where we know it is not normally present. This in turn, should improve the verifiability of predicted missing proteins. For example, if 40% of the predicted missing proteins were false positives due to complex families and the use of complex family information was able to eliminate these, the 20–25% recovery rate observed earlier in replicates would become 33–42%.

### Limitations of this study and future work

Our study demonstrates the potential of using networks for missing-protein prediction and offers strategical frameworks on how to recover these missing proteins using other supporting data, including gene expression, cross-replicate information, and peptide information. However, such strategies are purely inferential, and do not directly validate the existence of these missing proteins; in vitro validation of our FCS predictions would be necessary. Moreover, the aforementioned limitations of the protein complexome database and nature of proteome screen imposes limits on which proteins we can possibly predict or verify. The CORUM complex database was used as is, based on its recognized value from prior works. Nevertheless, future work can be performed to improve CORUM’s information value, and assess the impact of the network feature vector quality on performance. Based on our results, it may be particularly useful to expand the feature vector to include more complexes and generate tissue-specific network feature vectors, to see if further improvements to recovery and verification is attainable. There is also value in fine-tuning FCS parameters for better performance^19^.

## Conclusion

Recovery of missing proteins is a persistent problem that remains unsolved in proteomics. Using protein complexes and FCS, we have updated our analysis to include the latest DIA paradigm and designed a more sophisticated recovery-benchmarking scheme based on cross-batch replicates and proteins from the full PSM list.

## Supplementary Information

Supplementary Information.

## Data Availability

The datasets used and/or analyzed during the current study available from the corresponding author on reasonable request.

## Abbreviations

CR: Colorectal cancer
FCS: Functional class scoring
MPP: Missing protein problem
SWATH: Sequential window acquisition of all theoretical spectra
RC: Renal cancer
RCC: Renal cancer control
