# Supplementary material for: Resolving missing protein problems using functional class scoring
Source: Sci Rep. 2022 Jul 5;12:11358. doi: 10.1038/s41598-022-15314-3 (PMC9256666; doi:10.1038/s41598-022-15314-3)
Supplement: Supplementary file 1 — Supplementary Information. [file 41598_2022_15314_MOESM1_ESM.docx]

**Resolving missing protein problems using functional class scoring**

Bertrand Jernhan Wong^1,†^, Weijia Kong^1,†^, Limsoon Wong^2^, Wilson Wen Bin Goh^1,3,4,†,*^

1. School of Biological Sciences, Nanyang Technological University, Singapore

2. School of Computing, National University of Singapore, Singapore

3. Lee Kong Chian School of Medicine, Nanyang Technological University, Singapore

4. Center for Biomedical Informatics, Nanyang Technological University, Singapore

† These authors contributed equally

*Corresponding Author: Wilson Wen Bin Goh, wilsongoh@ntu.edu.sg

Address for correspondence/proofs:

Wilson Wen Bin Goh, PhD

School of Biological Sciences, Nanyang Technological University, 60 Nanyang Drive, Singapore, 637551

Lee Kong Chian School of Medicine, Nanyang Technological University, 60 Nanyang Drive, Singapore, 636921

Email: wilsongoh@ntu.edu.sg

**Supplementary Discussion**

**A summary of network-based tests for complex enrichment**

Network-based recovery of missing proteins typically leverages on the observation of *some*, but not necessarily *all* proteins within a biologically relevant network, to infer the presence of other network members that were undetected. In proteomic analyses, this is possibly due to instrumental issues or low sample input. These examples can be broadly categorized into three groups^1^.

The first is over-representation analysis, which tests the for statistically significant differences between differentially expressed proteins within complexes. One of the typical methods is the hypergeometric enrichment test (HE)^2^.

In HE, a t-statistic is calculated for each protein by comparing the expression of different groups. Then, the proteins are compared against the protein complexes. Given a total number of proteins N, M belongs to a complex with n differential proteins, the probability P that b or more proteins from the differential set are associated by chance with the complex is given by:

$$P\left( X\geq b \right)=\sum_{i=b}^{min\left( n,M \right)} \frac{\left( \begin{aligned} n \\ i \end{aligned} \right)\left( \begin{aligned} N-n \\ M-i \end{aligned} \right)}{\left( \begin{aligned} N \\ M \end{aligned} \right)}$$

P($X\geq b$) is the HE p-value. It is significant if it is smaller than alpha (typically 0.05).

The second type is direct-group analysis, which takes the protein complex as a whole and check its significance based on protein’s expression distribution. A representative method is Gene Set Enrichment Analysis (GSEA)^3^. GSEA is a Kolmogorov−Smirnov (KS) test-based algorithm. It works in the following manner: First, rank all proteins inside the sample. Then, KS is performed to test whether the rank of the specific protein inside and outside the reference complex conforms to the same distribution.

Specifically, by denoting proteins in the complex as the set C and proteins outside the complex as the set C’, the KS-statistic KS_C, C’_ is expressed as:

$${KS}_{C,C'}={max}_{x}\left| F_{1,C}\left( x \right)-F_{2,C'}\left( x \right) \right|$$

where $F_{1,C}\left( x \right)$ and $F_{2,C'}\left( x \right)$ are respectively the fraction of proteins in C and C’ whose rank is higher than the rank x.

Significance is computed using a theoretical Kolmogorov distribution as the null distribution. The null hypothesis is rejected at a significance threshold if

$${KS}_{C,C'}\geq c\left( alpha \right)*\sqrt{\frac{|C|+|C^{'}|}{|C| *|C^{'}|}}$$

where c(alpha) is the critical value at a given alpha level. At an alpha of 0.05, c(alpha) is set at 1.36.

The third type are network-based approaches. Examples include Proteomics Expansion Pipeline (PEP); Maxlink and Functional Class Scoring (FCS). In an earlier evaluation, FCS was shown to outperform PEP and Maxlink using the same complex feature space ^4,5^.

PEP is a cluster discovery-based approach, proteins are first filtered for differential candidates, denote as seed proteins. The protein list is then expanded based on a Protein-Protein Interaction Network (PPIN), where seed proteins are mapped to the PPIN, which is then expanded to include their first-degree neighbors. Analysis of overlapping cliques generates tightly connected clusters, such as via Palla’s Clique Percolation Method. Clusters are then scored and ranked:

$$S=\frac{\sum_{i=1}^{n} E_{i}}{n}$$

Where S is the calculated score, and E is the expression value for a detected protein

Maxlink is likewise an association-based approach. Suppose the PPIN, denote as G, contains set of nodes V and edges E. Like PEP, the proteins are preprocessed to classify the seed proteins. Among the set of seed proteins X ∈ V, and the non-seeds Y (Y = V − X), the set of linked proteins L is derived based on the regulation that the protein in Y has at least 2 connections to proteins in X, which is shown as the formula:

$$L =\{y \in Y |2 \leq|\{x \in X | (x,y) \in E\}|\}$$

The links are enumerated (i.e., the size of *L*) and associated proteins are and ranked.

FCS is a feature-based approach (see Methods, main text, for a detailed overview of the algorithm). Unlike PEP and Maxlink, which relies on PPINs and requirement proper threshold to select seed proteins. FCS does not require explicit topological information.

1. Goh, W. W. B. & Wong, L. Advancing Clinical Proteomics via Analysis Based on Biological Complexes: A Tale of Five Paradigms. *J. Proteome Res.* **15**, 3167–3179 (2016).

2. Goh, W. W. B., Lee, Y. H., Chung, M. & Wong, L. How advancement in biological network analysis methods empowers proteomics. *PROTEOMICS* **12**, 550–563 (2012).

3. Subramanian, A. *et al.* Gene set enrichment analysis: A knowledge-based approach for interpreting genome-wide expression profiles. *Proc. Natl. Acad. Sci.* **102**, 15545–15550 (2005).

4. Goh, W. W. B., Sergot, M. J., Sng, J. C. & Wong, L. Comparative Network-Based Recovery Analysis and Proteomic Profiling of Neurological Changes in Valproic Acid-Treated Mice. *J. Proteome Res.* **12**, 2116–2127 (2013).

5. Goh, W. W. B. & Wong, L. Advanced bioinformatics methods for practical applications in proteomics. *Brief. Bioinform.* **20**, 347–355 (2019).

# Supplementary Figures

**Supplementary Figure 1** **Abundance levels alone are not a sufficient explanation for missing proteins**. X-axis: Scaled mean expression values. Y-axis: Number of missing values across samples. The red hashed lines indicated the medians of each axis.

**Supplementary Figure 2** **Corresponding gene expressions of proteins specific to kidney and liver tissues tends to be discriminatory while proteins found in shared complexes have no such effects.**

**Supplementary Figure 3** **A:** **Recovery based on protein lists is very modest. B: Recovery based on shared FCS-predicted complexes is extremely high suggesting despite different protein lists, they point back to essentially similar complexes**

**
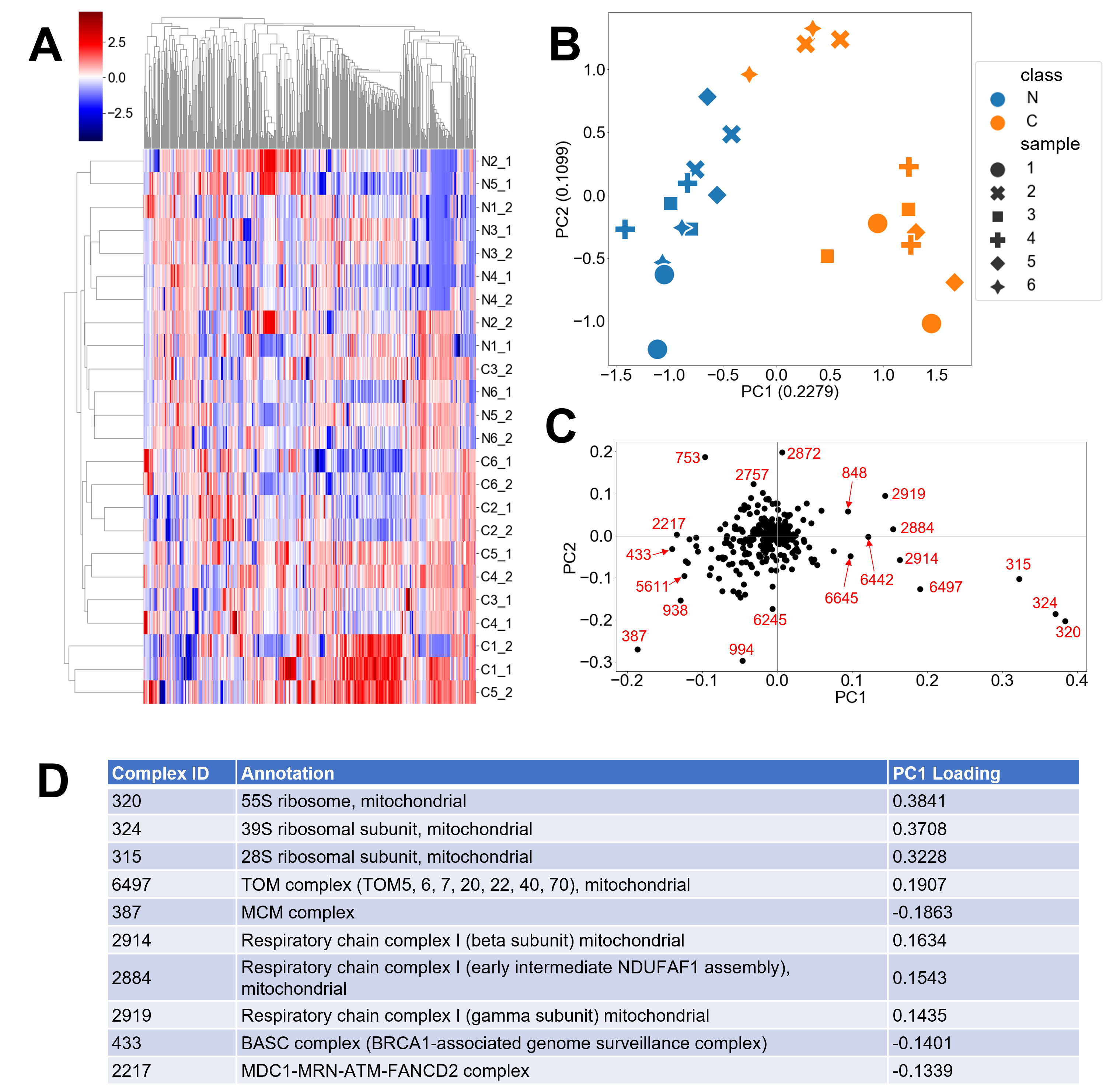
**

**Supplementary Figure 4: FCS p-values support discrimination of normal and cancer proteomes. A: Heatmap of Z-normalized FCS p-values, excluding complexes with 0 variance between samples. B: Principal component analysis (PCA) using FCS p-values shows noticeable separation of cancer and normal tissues. C: Loading plot highlights specifically enriched CORUM complexes by PC1 and PC2. D: Top 10 complexes by absolute PC1 loading, demonstrating high loading for ribosomal complexes.**
